# CircFGGY Inhibits Cell Growth, Invasion and Epithelial-Mesenchymal Transition of Hepatocellular Carcinoma *via* Regulating the miR-545-3p/Smad7 Axis

**DOI:** 10.3389/fcell.2022.850708

**Published:** 2022-05-03

**Authors:** Kun-Liang Feng, Na Diao, Zhai-Wen Zhou, Chong-Kai Fang, Ji-Nan Wang, Ying Zhang, Rui Luo, Chong Zhong

**Affiliations:** ^1^ Guangzhou University of Chinese Medicine, Guangzhou, China; ^2^ Department of Hepatobiliary Surgery, The First Affiliated Hospital of Guangzhou University of Chinese Medicine, Guangzhou, China; ^3^ Lingnan Medical Research Center, Guangzhou University of Chinese Medicine, Guangzhou, China; ^4^ Department of Gastroenterology, The Sixth Affiliated Hospital of Sun Yat-Sen University, Guangzhou, China; ^5^ Guangdong Provincial Key Laboratory of Colorectal and Pelvic Floor Diseases, Sixth Affiliated Hospital, Sun Yat-Sen University, Guangzhou, China; ^6^ Department of Radiology, The First Affiliated Hospital of Guangzhou University of Chinese Medicine, Guangzhou, China

**Keywords:** circFGGY, hepatocellular carcinoma, miR-545-3p, SMAD7, epithelial-mesenchymal transition

## Abstract

Hepatocellular carcinoma (HCC) is one of the leading causes of cancer-related death worldwide. Circular RNAs (circRNAs) play critical roles in the progression of HCC. However, the role of the newly identified circFGGY (hsa_circ_0006633) in the development and progression of HCC has not been explored. In this study, we found that circFGGY was significantly downregulated in tumor compared with that in adjacent normal liver tissues of patients with HCC. HCC patients with low circFGGY expression had poor overall survival after hepatectomy. Moreover, it was found that circFGGY could inhibit the proliferation, invasion and epithelial-mesenchymal transition of HCC both *in vivo* and *in vitro*. Mechanistically, circFGGY promoted the expression of Smad7, a well-known suppressor of the transforming growth factor-β signaling pathway. In addition, miR-545-3p, a tumor promoter targeting both circFGGY and Smad7, suppressed the upregulation of Smad7 caused by circFGGY overexpression. Collectively, our data revealed that circFGGY inhibits the proliferation and invasion of HCC cells by sponging miR-545-3p and promote the expression of Smad7, indicating that circFGGY functions as a tumor suppressor and could be a prognostic biomarker for HCC.

## Introduction

Primary liver cancer, as the third leading cause of cancer-related death around the world, has resulted in approximately 830, 000 deaths in 2020 (Sung et al., 2021). In spite of recent improvements in therapeutic measures, metastasis of liver cancer remains the largest single cause of mortality and the most difficult challenge in treatment. Due to the high rate of recurrence and metastasis, the 5-year survival rate of HCC is only about 14% (Li et al., 2016). As one of the most important clinical biomarkers of liver cancer, AFP is actually not a sensitive diagnostic biomarker (Benson et al., 2021). Farinati et al. confirmed the low sensitivity (54%) of AFP in the diagnosis of HCC in a clinical study (Farinati et al., 2006). It is still of paramount significance to investigate the pathogenesis and identify prognostic biomarkers and treatment targets for HCC.

Circular RNA (circRNA) is a new kind of non-coding RNA (ncRNA) with circular structure generated from back-splice events, in which the 3′ tail of a transcript is joined to the 5′ head of itself (Li et al., 2018). The vast majority of circRNAs consisted of exons and the length of most exonic circRNAs is between 200 and 1, 000 nucleotides (Yu et al., 2018). The unique circular structure makes them more stable than linear RNAs and resistant to the degradation by ribonuclease R (RNase R, a 3′ to 5′ exoribonuclease that digests linear RNAs) and with a longer half-life (Zhu et al., 2019). Owing to their high stability and tissue-specific expression, circRNAs are ideal candidates as liquid biopsy biomarkers. The level of circRNAs in body fluids was usually in consistent with that in tissues (Wang et al., 2021). In recent years, circRNAs have been reported to be associated with various cancers (Kristensen et al., 2018). Mechanistically, circRNAs often function as miRNA sponges with gene-regulatory potential (Huang et al., 2020). CircRNAs can also serve as scaffolds to enhance the interaction between target proteins (Wang et al., 2019; Li et al., 2021) or translatable transcripts that can produce protein isoforms (Liang et al., 2019; Jiang et al., 2021). MiRNA sponges are the most prevailing mechanism for the functions of circRNAs. Yu et al. demonstrated that cSMARCA5 inhibited the proliferation and migration of HCC by sponging miR-17-3p and miR-181b-5p, and promoting the expression of TIMP3 (Yu et al., 2018). Chen et al. showed that circGLIS2 promoted colorectal cancer cell motility by sponging miR-671 to activate the NF-κB signaling pathway (Chen et al., 2020). Despite that a large number of circRNAs have been identified, the biological functions of most circRNAs are yet to be illustrated.

Epithelial–mesenchymal transition (EMT) is a cellular process that epithelial cells lose cell-cell adhesion and acquire migratory and invasive capabilities (Du and Shim, 2016). EMT has been recognized as a cellular event that facilitates malignant transformation of tumors and a key step for tumor cells to gain high invasive and metastatic ability (Yang and Weinberg, 2008). In the EMT process, the expression of epithelial genes, such as E-cadherin and ZO-1, were deceased, and mesenchymal genes, such as N-cadherin and vimentin were increased. Thus, E-cadherin, N-cadherin and Vimentin often serve as biomarkers of EMT (Lamouille et al., 2014). Experimental studies have showed that EMT plays a pivotal role in HCC metastasis (Wang et al., 2017; Zhou et al., 2018). Several key signaling pathways have been shown to be closely associated with EMT, such as transforming growth factor beta (TGFβ), Wnt, Hedgehog and Notch (Gonzalez and Medici, 2014).

In this study, we firstly identified that circFGGY (hsa_circ_0006633), is downregulated in tumor tissue compared with that in adjacent normal liver tissues from HCC patients by high-throughput sequencing. CircFGGY is derived from FGGY gene, which encodes a protein that phosphorylates carbohydrates and played an important role in obesity, skeletal muscle atrophy and tumorigenesis (Taylor et al., 2018; Zhang et al., 2019; Smith et al., 2021). However, the role of circFGGY in HCC has not been elucidated. In the present study, we demonstrated that circFGGY inhibits the growth and invasion of HCC cells by inhibiting EMT. Mechanistically, circFGGY competitively binds to miR-545-3p to promote the expression of Smad7 and consequently suppress the motility of HCC cells. These results indicated that circFGGY functions as a potential tumor suppressor to regulate malignant transformation *via* circFGGY/miR-545-3p/Smad7 axis in HCC.

## Materials and Methods

### Clinical Tissues and Ethics Statement

In total, 56 pairs of HCC tumor and adjacent liver tissues were obtained from surgical resections of patients at the First Affiliated Hospital of Guangzhou University of Chinese Medicine (Guangzhou, China). Patients received no chemo- or radio-therapy prior to surgery. Among them, six pairs of tissues (Supplementary Table S1) were used for circRNA-sequencing. 50 pairs of tissues (Supplementary Table S2) were used for qRT-PCR validation and exploring the correlation between circFGGY expression and overall survival of patients after hepatectomy. The use of clinical samples and materials for research purposes were approved by the Ethics Committee of the First Affiliated Hospital of Guangzhou University of Chinese Medicine [ethical approval number ZYYECK (2019) 008].

### Quantitative Real-Time PCR

Total RNA was extracted using TRIzol Reagent (GLPBIO, Montclair, CA, United States). Reverse transcription for mRNA or circRNA was performed using Evo M-MLV RT Premix (Accurate Biology, Hunan, China) and cDNA amplification was performed using SYBR Green Premix Pro Taq HS qPCR Kit (Accurate Biology, China). Reverse transcription and cDNA amplification for miRNA were performed using miDETECT A Track miRNA qRT-PCR Starter Kit (RiboBio, Guangzhou, China) according to the manufacturer’s instructions. The primers are listed in Additional file: Supplementary Table S3.

### Western Blot

The total protein of HepG2 and MHCC97H cells was extracted using RIPA lysis buffer (Beyotime, Shanghai, China), and equal amounts of protein lysates were separated on 10% SDS-PAGE gels. The separated protein bands were transferred onto polyvinylidene fluoride membrane. The membranes were incubated overnight at 4°C with the following primary rabbit-antibodies: Smad7 (1:1,000), E-cadherin (1:5,000), N-cadherin (1:5,000), Vimentin (1:5,000), GAPDH (1:5,000) and β-actin (1:2000) (Proteintech, Wuhan, China). After washing with TBST (15 min × 3), the membranes were incubated for 1.5 h at room temperature with anti-rabbit IgG, HRP-linked secondary antibody (1:3,000, Cell Signaling Technology, MA, United States). Then, the membranes were washed with TBST again (10 min × 4). Finally, the immunoreactive bands were visualized using the Immobilon® Western Chemiluminescent HRP Substrate (Millipore, Billerica, MA, United States).

### RNase R and Actinomycin D Treatment

HepG2 cells were planted into six-well plates (3 × 10^5^ cells/well). After 24 h, cells were treated with 5 μg/ml Actinomycin D (Cayman Chemical, Ann Arbor, MI, United States) or DMSO and collected at indicated time points. Total RNA (2 μg) was incubated with 3 U/μg of RNase R (Geneseed, Guangzhou, China) for 20 min at 37°C and 10 min at 70°C. After treatment with Actinomycin D or RNase R, the expression of circFGGY and FGGY mRNA were analyzed by qRT-PCR.

### RNA Fluorescence *in Situ* Hybridization

The probe for circFGGY was 5′-cy3-AGATTAGAACACTTTCCCGCATCC-3′. Fluorescent *In Situ* Hybridization Kit (RiboBio, China) was used in this assay. The HepG2 cells were seeded on the cell culture slide within 24-well plate (3 × 10^4^ cells/well). After 24 h incubation, the cells were fixed with 4% paraformaldehyde, hybridized with circFGGY-cy3 probes and the nuclei were stained with DAPI staining solution according to the manufacturer’s instructions. The images were acquired using a confocal microscope (Leica TCS SPE Ⅱ, Wetzlar, Germany).

### Silencing and Overexpressing of CircFGGY

The circFGGY-specific-ASO was designed and synthesized by RiboBio Co., Ltd. (Guangzhou, China). The overexpression plasmid of circFGGY was constructed by Shanghai Lianfeng Co., Ltd. (Shanghai, China).

### Cell Proliferation Assay

The cell proliferation ability was assessed by cell counting kit-8 (CCK-8) assay and 5-Ethynyl-20-deoxyuridine (EdU) assay. The transfected cells were plated in a 96-well plate (4,000 cells/well). Then, on the indicated day, 10 μl of CCK-8 reagent (GLPBIO, United States) was added directly to the culture medium. After incubating for 1.5 h at 37°C, the absorbance (450 nm) was measured by Spectrophotometer (Thermo Fisher Scientific, Waltham, MA, United States).

EdU assays were performed using a Cell-Light EdU *In Vitro* Kit (RiboBio, China) according to the manufacturer’s instructions. The transfected cells were plated in a 24-well plate (5 × 10^4^ cells/well). Then, the cells were incubated with 50 μM EdU for 2 h, and stained with ApolloDye Solution and Hoechst33342. The cell proliferative ability was assessed based on the percentage of Edu-positive cells.

### Transwell Invasion Assay

The diluted Matrigel Basement Membrane Matrix (50 μg/100 μl, Corning, Glendale, Arizona, United States) was added to the upper surfaces of transwell inserts (Corning, United States) and incubated at room temperature for 1 h. Transfected cells (1 × 10^5^) in 100 μl of serum-free DMEM medium were added to the inside of the transwell inserts chamber. A total of 600 μl of DMEM medium supplemented with 10% FBS was added into the lower chamber. After incubation for 24 h, the cells that did not invade were scraped off and invaded cells were fixed with 4% paraformaldehyde and stained with 0.5% crystal violet solution (Beyotime, China). The invasive ability of cells was assessed based on the number of the invaded cells.

### Dual-Luciferase Reporter Assay

PmirGLO-SMAD7 and pmirGLO-circFGGY Dual-Luciferase Reporter Vector (HedgehogBio, Shanghai, China) was constructed. The mutant strand was mutated in the putative binding site for miR-545-3p in SMAD7 3′-UTR region and circFGGY sequence. The wild-type (WT) or mutant SMAD7/circFGGY luciferase reporter plasmids (1 μg/ml) were co-transfected into MHCC97H cells with miR-545-3p mimics or NC-mimics (100 nM), respectively. Dual Luciferase Reporter Gene Assay Kit (Beyotime, China) was applied to assess the Firefly and Renilla luciferase activity 24 h after transfection. Data were acquired using the Multilabel Reader (PerkinElmer, Waltham, MA, United States).

### Animal Study

The research was conducted in accordance with the internationally accepted principles for laboratory animal use and care as found in the Guideline for the Care and Use of Laboratory Animals published by the National Institutes of Health (NIH publication #85–23, revised in 1985). All animal procedures were approved by the Guangzhou University of Chinese Medicine Animal Ethics Committee (animal’s review number is: 20210721005). The five-weeks-old male BALB/c nude mice were purchased from Guangdong Medical Experimental Animal Center (Guangzhou, China), housed under Specific Pathogen Free Experimental Animal Center of Guangzhou University of Chinese Medicine. MHCC97H cells with circFGGY stably overexpressed or the NC cells (5 × 10^6^ cells in 0.2 ml of PBS) were injected to the axillary region on the right side (*n* = 5/group).

After 2 weeks, the circFGGY overexpressed groups were subcutaneously injected with miR-545-3p agomir or NC-agomir (Designed and synthesized by RiboBio). After 4 weeks, the mice were sacrificed, and the xenograft tumors were excised and analyzed by immunohistochemistry and hematoxylin and eosin (HE) staining. For imaging tumors in live animals, mice were anaesthetized with 2% isoflurane and injected intraperitoneally with 100 µl of the D-Luciferin sodium solution (15 mg/ml, MedChemExpress, NJ, United States). After 10 min, images were acquired with the *in vivo* imaging system (NightOwl II LB 983, Berthold, Germany).

### Statistical Analysis

GraphPad Prism version 8 (GraphPad Software, San Diego, CA) or SPSS 19 (SPSS Inc., Chicago, IL, United States) was used for data analysis. The Quantitative data were expressed as mean ± standard deviation (SD). Qualitative data were expressed by ratios. Student’s t test, chi-squared test, one-way ANOVA, two-way ANOVA, Wilcoxon signed-rank test, Pearson correlation analysis and log-rank test were performed, depending on the purpose, data types and grouping. In addition, Dunnett’s multiple comparisons test was performed after one way ANOVA. The selection of statistical methods is described in the figure legends. Statistical significance was indicated by a *p* value less than 0.05 (*p < 0.05, **p < 0.01, ***p < 0.001). All experiments were repeated for at least three times.

## Results

### CircFGGY Is Downregulated in Human Hepatocellular Carcinoma Tissues and Cells

**FIGURE 1:**
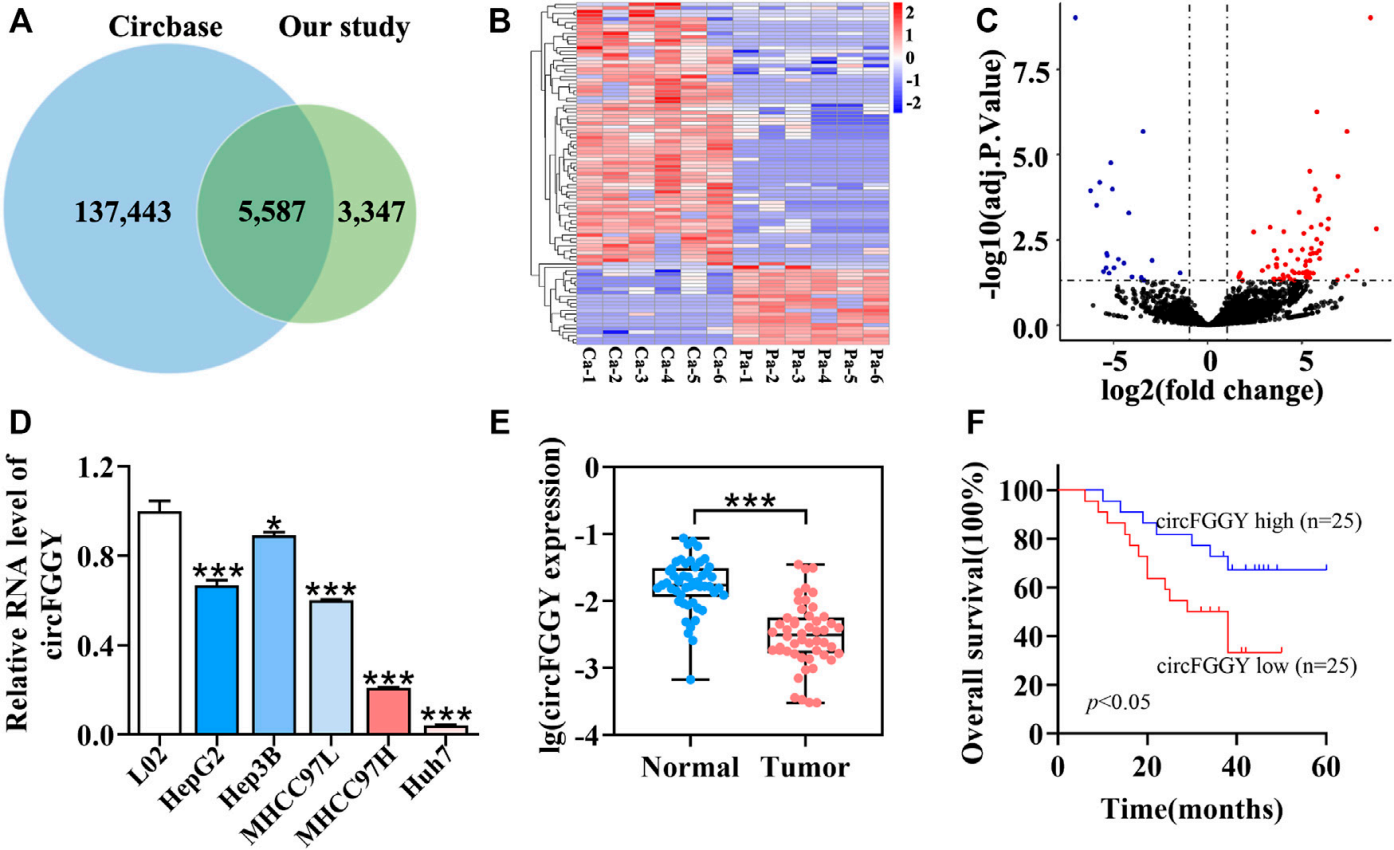
Identification and characteristics of circFGGY. **(A)** Venn diagram showing the intersection between circRNAs identified in our study and circBase. **(B)** Clustered heatmap of the differentially expressed circRNAs in six paired HCC and adjacent normal tissues. Rows represent circRNAs while columns represent tissues. **(C)** Volcano plots showing the identification of circRNAs. The red color indicates upregulated genes (adj P. value < 0.05, log 2 FC > 1) and blue color indicates downregulated genes (adj P. value < 0.05, log 2 FC < -1), while black color indicates genes with no significant differences among the HCC and adjacent normal tissues. Log2 FC means that log2 transformation to fold changes of circRNA expression between HCC tissues and adjacent normal tissues. **(D)** qRT-PCR comparing the expression of circFGGY in various HCC cell lines and the normal liver cell line L02. One-Way ANOVA was used. Data are represented as mean ± SD, *n* = 3. Comparing with LO2, *: *p* < 0.05; ***: *p* < 0.001. **(E)** Analysis of RNA expression of circFGGY in the 50 paired HCC and adjacent normal tissues using qRT-PCR. Wilcoxon matched-pairs signed rank test was used. Data represented as median (Min, Max), *n* = 50. ***: *p* < 0.001. **(F)** Kaplan-Meier analysis of the association of circFGGY expression and the OS of HCC patients after hepatectomy. Log-rank test was used, *n* = 50.

To examine the expression of circRNAs in HCC, six pairs of human HCC clinical samples and adjacent normal tissues were analyzed using circRNA microarray assays. A total of 8,934 distinct circRNAs were detected. Among them, 5,587 circRNAs have been recorded in circBase (Glazar et al., 2014) (Figure 1A). As the heatmap (Figure 1B) and volcano plot (Figure 1C) showed, 95 circRNAs were differentially expressed in tumor tissues and adjacent normal tissues. Among them, 73 circRNAs were upregulated (adjusted *p* value < 0.05, lgFC ≥ 1) while 22 were downregulated (adjusted *p* value < 0.05, lgFC ≤ -1).

It was found that circFGGY was downregulated in the HCC tumor samples compared with the adjacent normal tissues, which has not been reported previously. The expression of circFGGY was validated in various HCC cell lines and HCC clinical samples. The expression of circFGGY was significantly decreased in HCC cell lines including HepG2, Hep3B, MHCC97L, MHCC97H and Huh7 compared to that of the normal liver cell line L02 (Figure 1D). To confirm our findings, the level of circFGGY was quantified in 50 paired HCC and adjacent normal tissues by qRT-PCR (Figure 1E). In line with the RNA-seq results, the level of circFGGY was dramatically lower in tumor tissues than that in adjacent normal tissues. The Kaplan-Meier’s survival curves revealed that the HCC patients with low circFGGY expression had poor overall survival [(OS) *p* < 0.05] after hepatectomy (Figure 1F). Furthermore, the correlations between circFGGY expression and other clinical characteristics of 50 patients with HCC were analyzed (Table 1). BCLC stage and Edmondson’s grade were correlated with the expression of circFGGY. These results revealed that CircFGGY is downregulated in human hepatocellular carcinoma tissues and cells and might be associated with the pathogenesis of HCC.

**TABLE 1:** Association between circFGGY expression and clinical characteristics of hepatocellular carcinoma patients.

Variable	circFGGY		*p* value
	Low	High	
All cases	25	25	
Age, years, >50: ≤50	15:10	15:10	1.000
Gender, male/female	19:6	18:7	1.000
Tumour size, cm, >5: ≤5	13:12	9:16	0.393
TNM stage, T2+T3:T1	13:12	7:18	0.148
BCLC stage, B + C: A	12:13	4:21	**0.032**
vascular invasion, present: absent	5:20	2:23	0.463
Edmondson’s grade, III + IV: I + II	16:9	10:15	**0.046**
Liver cirrhosis, with/without	7:18	10:15	0.551
No. tumour, multiple: solitary	9:16	5:20	0.345

p value < 0.05 is shown in bold.

### Cellular Localization and Stability of circFGGY in Hepatocellular Carcinoma Cells

**FIGURE 2:**
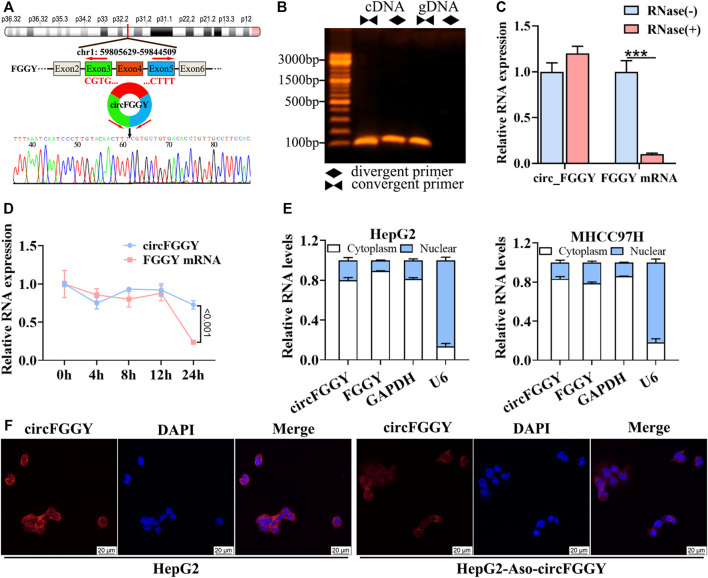
Cellular localization and stability of circFGGY in HCC cells. **(A)** Schematics showing the generation of circFGGY. PCR primers used to specifically detect circFGGY by qRT-PCR are indicated by red arrows. Black arrow indicates regions that were Sanger-sequenced and identified as the breakpoint of circFGGY. **(B)** CircFGGY can be amplified by the divergent primers in cDNA, but not in gDNA. **(C,D)** qRT–PCR analysis for the RNA expression of circFGGY and FGGY after treatment with RNase R or Actinomycin D (5 ug/ml) at the indicated time points in HepG2 cells. Data were shown as mean ± SD, *n* = 3. ***p < 0.001 in independent Student’s t test **(C)** or two-way ANOVA **(D)**. **(E)** circFGGY and FGGY are abundant in the cytoplasm of HepG2 and MHCC97Hcells. GAPDH and U6 were applied as positive controls in the cytoplasm and nucleus, respectively. **(F)** *In situ* hybridization showing the subcellular localization of circFGGY in HepG2 cells. The circFGGY probe was labeled with Cy3. Nuclei were stained with DAPI. Scale bar, 20 µm.

To confirm the circular structure and characteristics of circFGGY, we firstly designed a pair of divergent primers that were specific for circFGGY and a pair convergent primer for FGGY mRNA. According to circBase, circFGGY was consisted of FGGY exons 3–5 and the specific back splicing site of circFGGY were examined by Sanger sequencing (Figure 2A). The PCR for cDNA and gDNA template using the divergent and convergent primers were performed respectively. The results showed that FGGY was amplified from the two kinds of DNA template, while circFGGY only existed in the cDNA template (Figure 2B).

Moreover, circFGGY was tolerated to RNase R, indicating that circFGGY is a circular RNA (Figure 2C). Furthermore, the half-life of circFGGY and FGGY mRNA in HepG2 cells treated with actinomycin D (5 ug/ml), a transcription inhibitor, was evaluated. The result showed that circFGGY was more stable than FGGY mRNA (Figure 2D). In addition, the results of qRT-PCR and *in situ* hybridization against circFGGY showed that circFGGY mainly distributed in the cytoplasm (Figures 2E,F). In brief, circFGGY was a circular and stable transcript that was generated from back-splicing of the FGGY gene transcript.

### CircFGGY Inhibits Cell Growth, Migration and Invasion of HCC Both *In Vitro* and *In Vivo*

**FIGURE 3:**
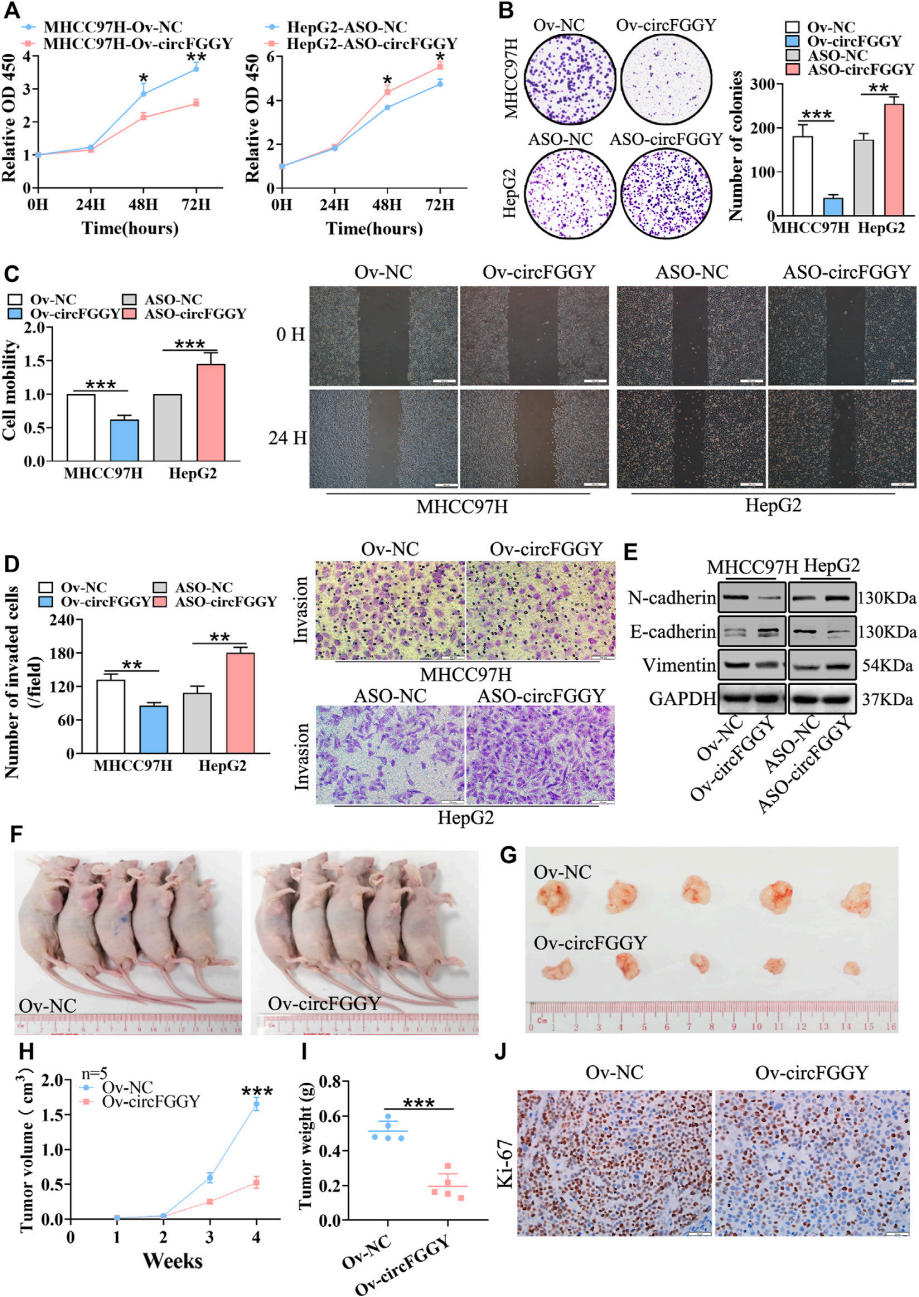
CircFGGY inhibits HCC growth, migration, invasion and EMT both *in vitro* and vivo. A-D. CCk8 assays **(A)**, colony formation assays **(B)**, scratch wound healing assay **(C)** and transwell invasion assay **(D)** assessed the cell proliferation, colony formation, migration and invasion potential in MHCC97H cells transfected with the circFGGY overexpression vector and HepG2 cells transfected with the circFGGY ASO (*n* = 3). Scale bar, 500 µm **(C)**, 100 µm **(D)**. **(E)** Western blot detected the expression of the EMT biomarkers in MHCC97H cells transfected with circFGGY overexpression vector and HepG2 cells transfected with ASO-circFGGY. **(F)** Nude mice inoculated subcutaneously with the circFGGY-overexpressing MHCC97H cells or NC cells. **(G)** Representative images of the MHCC97H xenograft tumors after 4 weeks of tumor growth in the circFGGY overexpression and NC groups. **(H,I)** The volume and weight of subcutaneous xenograft tumors (*n* = 5). **(J)** IHC analysis on Ki-67 expression in xenograft tumor tissues from the circFGGY overexpression and NC groups. Scale bar, 50 µm. Data were shown as mean ± SD. *p < 0.05, **p < 0.01, ***p < 0.001 in two-way ANOVA **(A,H)** or independent Student’s t test **(B,C,D,I)**.

To investigate the biological functions of circFGGY in HCC, we conducted a series of experiments both *in vitro* and *in vivo*. Firstly, we constructed a circFGGY-overexpression plasmid and anti-sense oligonucleotide (ASO) targeting circFGGY specific back splicing sequence. According to the expression of circFGGY in various HCC cell lines, MHCC97H with low expression of circFGGY was transfected with circFGGY-overexpression plasmid, while HepG2 with high expression of circFGGY was transfected with circFGGY-ASO (Supplementary Figure S1A). We successfully upregulated the expression of circFGGY in MHCC97H cells and knocked down the expression of circFGGY in HepG2 cells (Supplementary Figure S1B) Then, the circFGGY-transfected cells were subjected to CCK8 assays, colony-formation assays, wound healing assays and transwell invasion assays to respectively test their cell growth, colony formation, migration and invasion properties. As expected, overexpression of circFGGY led to inhibition of growth, colony formation, migration and invasion in MHCC97H cells. In contrast, circFGGY silence promoted the cell growth, colony formation, migration and invasion in HepG2 cells (Figures 3A–D).

As showed above, circFGGY inhibited the growth and invasion of HCC cells. Therefore, we examined the expression of the biomarkers of EMT in HCC cells transfected with circFGGY overexpression vector or ASO-circFGGY. The results showed that circFGGY significantly promoted the expression of E-cadherin and decreased the expression of N-cadherin and Vimentin, indicating the inhibition of EMT (Figure 3E and Supplementary Figure S1C).

To further confirm the role of circFGGY *in vivo*, MHCC97H cells were stably transduced with lentiviral vectors containing circFGGY sequence. Subcutaneous injection of circFGGY–overexpressing MHCC97H cells showed decreased tumor growth rate. Strikingly, the tumor volume and weight at endpoint in mice of Ov-circFGGY group were about 0.52 ± 0.17 cm^3^ and 0.20 ± 0.07 g respectively, significantly lower than that in mice of NC-group which were about 1.66 ± 0.18 cm^3^ and 0.50 ± 0.05 g (Figures 3F–I). IHC analyses showed that the Ki-67 expression was dramatically decreased after overexpressing circFGGY (Figure 3J and Supplementary Figure S1D). These results suggested that circFGGY was involved in the regulation of cell growth, invasion and EMT in HCC.

### CircFGGY Promotes the Expression of Smad7 by Competitively Targeting miR-545-3p

**FIGURE 4:**
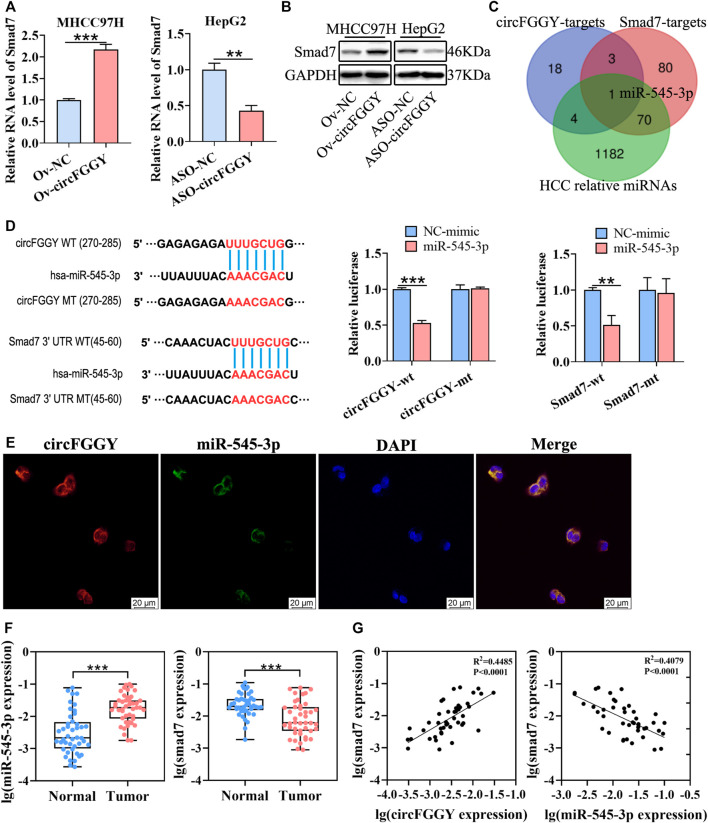
CircFGGY promotes the expression of Smad7 by competitively targeting miR-545-3p. **(A,B)** qRT-PCR and Western blot detected the RNA and protein expression of Smad7 in MHCC97H cells transfected with circFGGY overexpression vector and HepG2 cells transfected with ASO-circFGGY. **(A)**: data were shown as mean ± SD, *n* = 3. **p < 0.01, ***p < 0.001 in independent Student’s t test. **(C)** Venn diagram showing potential miRNA targets for circFGGY and SMAD7. The miRNA targets for circFGGY were obtained from circbank database. The miRNA targets for Smad7 were obtained from targetscan and miRDB database. The HCC associated miRNAs were obtained from MNDR database. **(D)** The luciferase activity of the wild-type or mutant circFGGY/Smad7 after co-transfection with miR-545-3p mimics or NC-mimics in MHCC97H cells. Data were shown as mean ± SD, *n* = 3. **p < 0.01, ***p < 0.001 in independent Student’s t test. Wt, wild-type; mt, mutant. **(E)** *In situ* hybridization assay showing colocalization of endogenously expressed circFGGY and miR-545-3p in the cytoplasm. The circFGGY probes were labeled with cy3. The miR-545-3p probes were labeled with FITC. Nuclei were stained with DAPI. Scale bar, 20 µm. **(F)** qRT-PCR detected the RNA expression of miR-545-3p and Smad7 in the 50 paired HCC and adjacent normal tissues. Wilcoxon matched-pairs signed rank test was used. Data represented as median (Min, Max), *n* = 50. ***: *p* < 0.001. **(G)** Pearson correlation analysis between RNA expression of Smad7 and circFGGY/miR-545-3p in clinical tumor tissues. *n* = 50.

It is well known that the TGFβ signaling pathway plays a critical role in the process of EMT. We investigated the RNA expression of the important molecule in the TGFβ signaling pathway. We found that circFGGY regulated RNA expression of Smad7 (Figure 4A) but not smad2, smad3, smad4, smad6 or FGGY, the host gene of circFGGY (Supplementary Figure S2A). The protein expression of Smad7 was also significantly increased in the cells overexpressed circFGGY and decreased in the cells with silence of circFGGY (Figure 4B, Supplementary Figure S2B).

As previously reported, circRNAs primarily functioned as miRNA sponges to regulate gene expression (Yu et al., 2018; Huang et al., 2020; Ren et al., 2021). We tried to find out the potential common miRNA target for Smad7 and circFGGY, which is associated with liver cancer. Through the Venn diagram analysis, it was found that miR-545-3p was the potential target (Figure 4C). To confirm this prediction, a dual-luciferase assay was performed. Targetscan (http://www.targetscan.org/vert_72/) was used to predict the binding sites of miR-545-3p and circFGGY/Smad7. miR-545-3p mimics or NC mimics were co-transfected with the circFGGY or Smad7-3′UTR luciferase reporters into the MHCC97H cells. Compared with the NC-mimics, miR-545-3p significantly reduced the luciferase reporter activity in the wild-type group of circFGGY and Smad7, but the luciferase activity did not significantly change after the target sites of miR-545-3p were mutated (Figure 4D). By performing *in situ* hybridization assays, we confirmed that circFGGY was colocalized with miR-545-3p in the cytoplasm (Figure 4E).

To explore the relation between Smad7 and circFGGY/miR-545-3p, we examined the RNA expression of miR-545-3p and Smad7 in the 50 paired HCC and adjacent normal tissues. Quantitative RT-PCR analysis showed that the miR-545-3p expression was higher, while the Smad7 expression was significantly lower in the HCC tissues than that in the adjacent normal tissues of these 50 patients with HCC (Figure 4F). Through Pearson correlation coefficients analysis, we found that the mRNA expression of Smad7 was positively correlated with the expression of circFGGY and negatively correlated with the expression of miR-545-3p (Figure 4G).

Moreover, miR-545-3p did not show significant changes after overexpressing or silencing of circFGGY (Supplementary Figure S2C). Similarly, circFGGY did not show significant changes after overexpression or knock-down of miR-545-3p (Supplementary Figure S2D). These findings suggested that circFGGY and miR-545-3p was not abolished by each other. Collectively, our data supported that circFGGY functions as a sponge of miR-545-3p, and upregulates the expression of Smad7.

### miR-545-3p Promotes Hepatocellular Carcinoma Growth, Migration and Invasion

**FIGURE 5:**
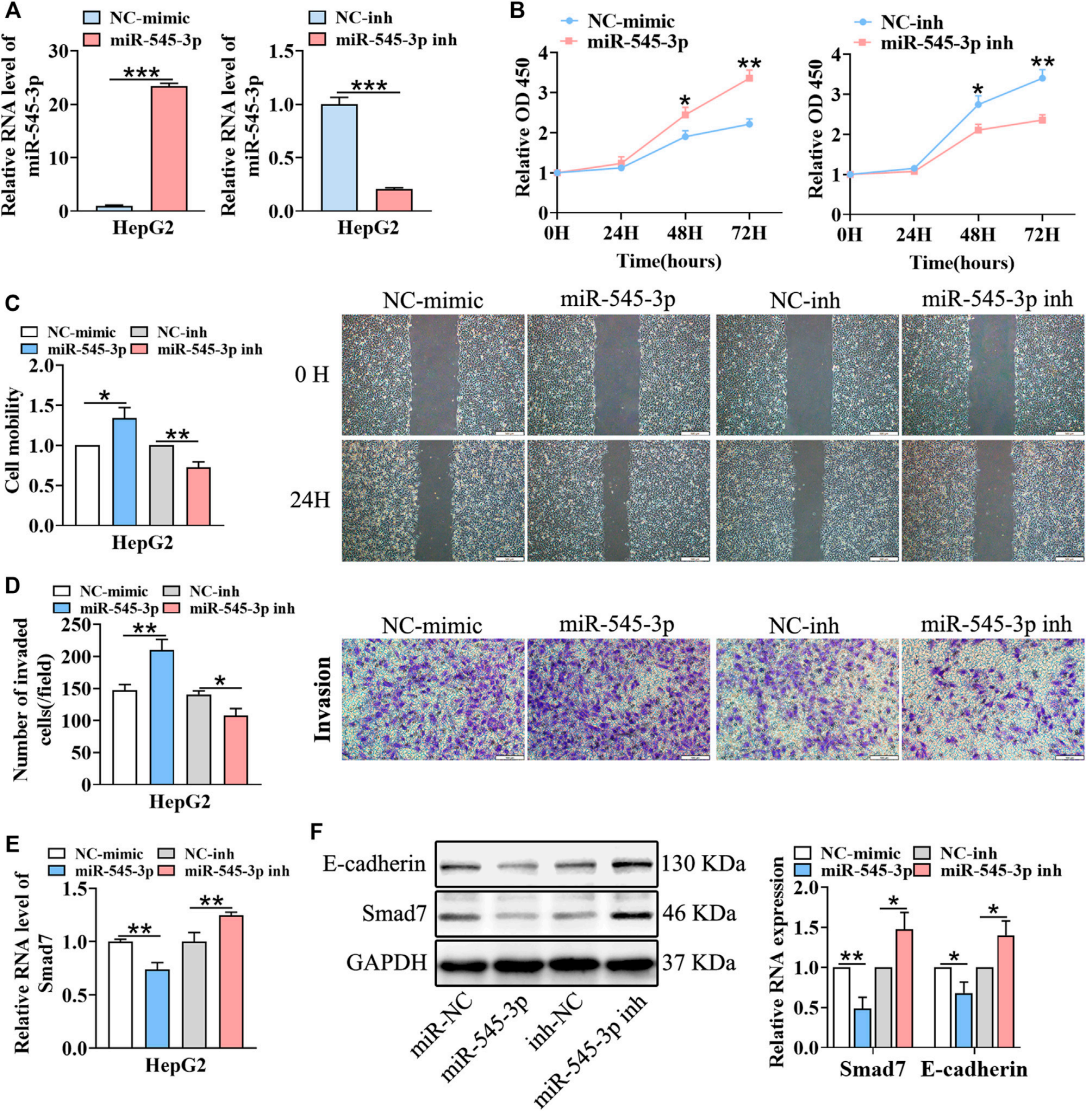
miR-545-3p promotes cell growth, migration, invasion, EMT and downregulates the expression of Smad7 *in vitro*. **(A)** qRT-PCR validated miR-545-3p upregulation in HepG2 cells transfected with miR-545-3p mimics and downregulation in HepG2 cells transfected with miR-545-3p inhibitors. B-D. CCk8 assays **(B)**, scratch wound healing assay **(C)** and transwell invasion assay **(D)** assessed the cell proliferation, migration and invasion potential in HepG2 cells transfected with miR-545-3p mimics or inhibitors. Scale bar, 500 µm **(C)**, 100 µm **(D)**. **(E)** qRT-PCR detected the RNA expression of Smad7 in HepG2 cells transfected with miR-545-3p mimics or inhibitors. **(F)** Western blot detected the expression of Smad7 and E-cadherin in HepG2 cells transfected with miR-545-3p mimics or inhibitors. Data were shown as mean ± SD, (*n* = 3). *p < 0.05, **p < 0.01, ***p < 0.001 in independent Student’s t test **(A,C–F)** and in two-way ANOVA **(B)**.

According to a previous report, miR-545-3p was a tumor promoter in HCC (Changjun et al., 2018). Thus, we hypothesized that circFGGY inhibited the proliferation and metastasis of HCC by protecting Smad7 from digestion by miR-545-3p in HCC. To test this hypothesis, we upregulated and downregulated the expression of miR-545-3p through transfecting miR-545-3p mimics or inhibitors (Figure 5A). Then, the proliferation, migration and invasion of cells, as well as the expression of E-cadherin and Smad7 were assessed. As expected, the proliferation, migration and invasiveness of cells were increased in HepG2 transfected with the miR-545-3p mimics and decreased in HepG2 transfected with the miR-545-3p inhibitors, compared with the NC-group (Figures 5B–D). The mRNA and protein expression of Smad7, as well as the protein expression of E-cadherin were downregulated upon overexpression of miR-545-3p mimics and upregulated upon knock-down of miR-545-3p (Figures 5E,F).

### CircFGGY Inhibits the Growth and Metastasis of Hepatocellular Carcinoma Through the miR-545-3p/Smad7 Axis *In Vitro*

**FIGURE 6:**
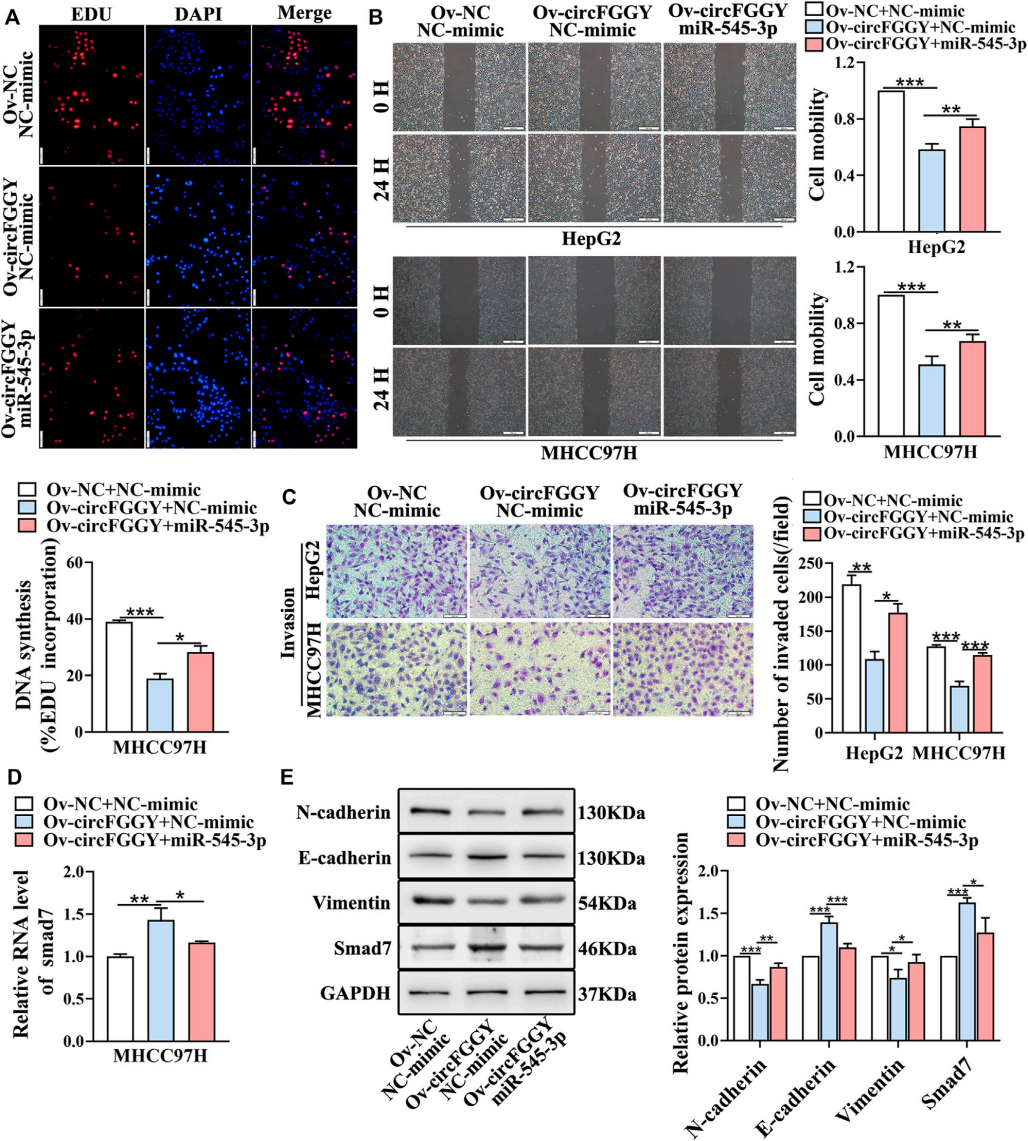
CircFGGY inhibits the growth and metastasis of HCC through the miR-545-3p/Smad7 axis *in vitro*. **(A–C)** The cell proliferation **(A)**, migration **(B)** and invasion **(C)** decreased after the overexpression of circFGGY, and these processes were reversed after overexpression of miR-545-3p in MHCC97H or HepG2 cells. Scale bar, 500 µm **(B)**, 100 µm **(A,C)**. **(D)** The mRNA expression of Smad7 was upregulated after overexpression of circFGGY, and the expression was reversed after overexpression of miR-545-3p in MHCC97H cells. **(E)** Western blot detected the protein expression of Smad7 and the biomarkers of EMT after the overexpression of circFGGY or circFGGY/miR-545-3p in MHCC97H cells. One-Way ANOVA was used **(A–E)**. Data are represented as mean ± SD, *n* = 3. Comparing with Ov-circFGGY + NC-mimic group, *: *p* < 0.05; **: *p* < 0.01; ***: *p* < 0.001.

We next explored whether circFGGY inhibits the invasiveness of HCC by sponging miR-545-3p. Functional experiments showed that the overexpression of miR-545-3p alleviated the inhibition of cell proliferation migration, and invasion of HCC cells caused by circFGGY overexpression (Figures 6A–C). Meanwhile, the mechanism-related experiments revealed that circFGGY promoted the mRNA and protein expression of Smad7 and reduced the level of EMT biomarkers, and the expression of these molecules was rescued by overexpression of miR-545-3p (Figures 6D,E).

### CircFGGY Inhibits the Growth of Hepatocellular Carcinoma by Regulating the miR-545-3p/Smad7 Axis *In Vivo*

**FIGURE 7:**
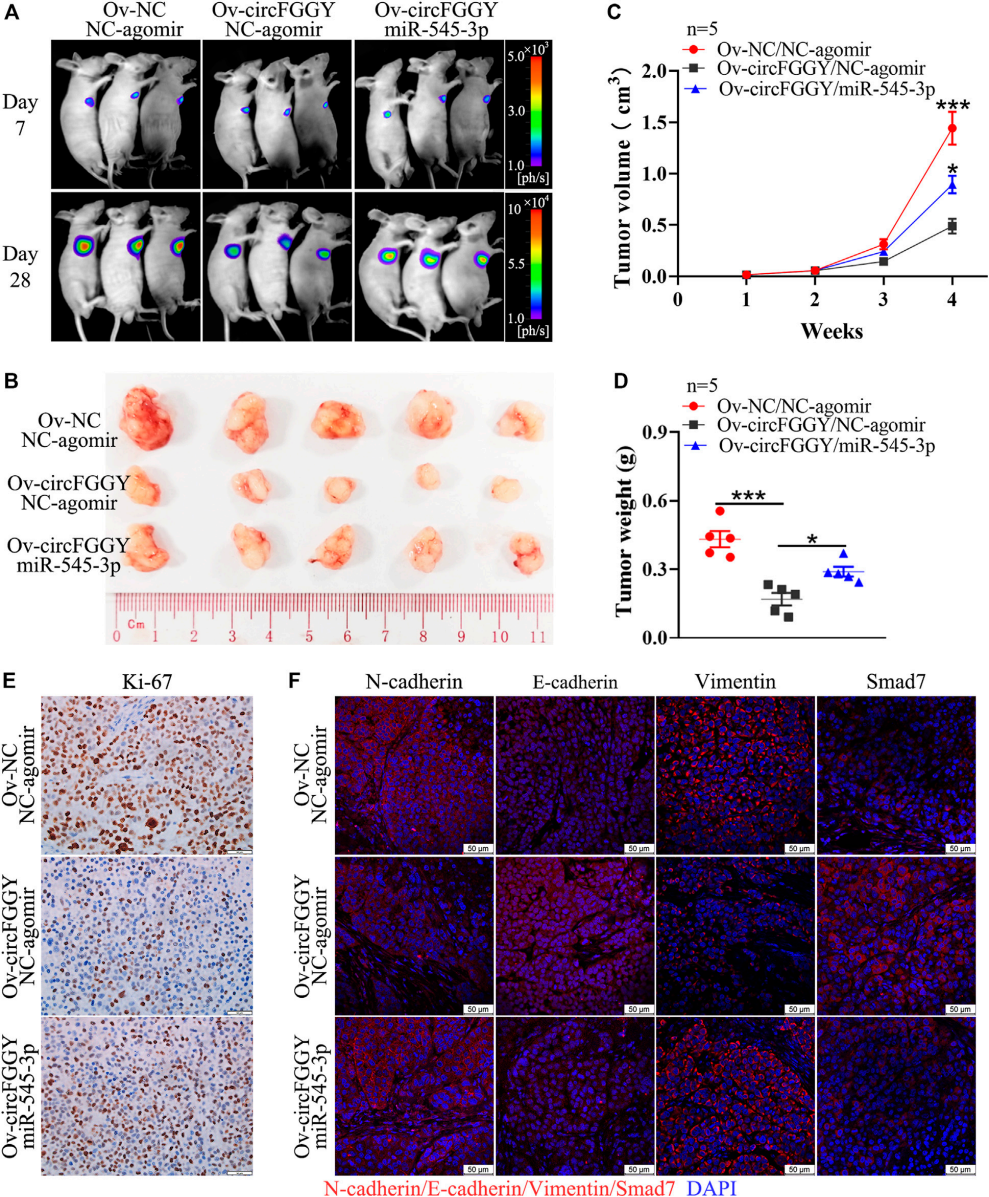
CircFGGY inhibits the growth of HCC through the miR-545-3p/Smad7 axis *in vivo*. **(A)**. Representative bioluminescence images of xenograft tumors in mice at day 7 and day 28 after injection of MHCC97H cells. **(B)**. Representative images of the MHCC97H xenograft tumors after 4 weeks of tumor growth in circFGGY overexpression, circFGGY/miR-545-3p overexpression and NC group. **(C,D)** The volume and weight of subcutaneous xenograft tumors. **(E)** IHC analysis of Ki-67 expression in circFGGY overexpression, circFGGY/miR-545-3p overexpression and NC group. Scale bar, 50 µm. **(F)** Immunofluorescence (IF) analysis of EMT biomarkers and Smad7 expression in xenograft tumor tissues from the circFGGY overexpression, circFGGY/miR-545-3p overexpression and NC groups. Scale bar, 50 µm. NC group: mice were subcutaneous injected with NC-overexpressing MHCC97H cells and NC-agomir was injected around the xenograft tumor every 4 days from the second week. CircFGGY overexpression group: mice were subcutaneous injected with circFGGY-overexpressing MHCC97H cells and NC- agomir was injected around the xenograft tumor. CircFGGY/miR-545-3p overexpression group: mice were subcutaneous injected with circFGGY-overexpressing MHCC97H cells and miR-545-3p agomir was injected around the xenograft tumor. Data are presented as mean ± SEM, *n* = 5/group. *p* values were calculated by two-Way ANOVA **(C)** and One-Way ANOVA **(D)**.

To further explore the influence of circFGGY/miR-545-3p/Smad7 axis in tumor growth *in vivo*, we established a mouse HCC model. It was found that the suppression of xenograft tumor growth by overexpressing circFGGY was reversed by miR-545-3p agomir (Figures 7A–D). In line with *in vitro* study, the expression of E-cadherin and Smad7 was dramatically increased and that of Ki-67, N-cadherin and Vimentin was significantly decreased in the xenograft tumor with overexpression of circFGGY, which could be rescued by miR-545-3p agomir (Figures 7E,F and Supplementary Figure S2E).

Collectively, these observations demonstrate that circFGGY inhibits cell growth, invasion and EMT of HCC *via* regulating the miR-545-3p/Smad7 axis.

## Discussion

In recent years, circRNAs have attracted great interest in cancer research. An increasing number of circRNAs have been reported to play important regulatory roles in various tumors. As reported, circNSUN2, circGLIS2 and circLONP2 can enhance colorectal cancer progression (Chen et al., 2019; Chen et al., 2020; Han et al., 2020). CircYAP1 can suppress the proliferation and invasion of gastric cancer (Wu N et al., 2021), circNDUFB2 inhibits non-small cell lung cancer progression (Li et al., 2021) and circ-DNMT1 functions as a facilitator in breast cancer (Du et al., 2018). CircPRRC2A enhances angiogenesis and metastasis through EMT in renal cell carcinoma (Li et al., 2020). Furthermore, circASAP1, circ-CDYL and hsa_circ_0058493 function as oncogenes in HCC(Hu et al., 2020; Wei et al., 2020; Wu A et al., 2021), while cSMARCA5 serves as a tumor suppressor (Yu et al., 2018). The circRNAs reported above have been shown to be associated with the prognosis of various cancers and may serve as prognostic biomarkers.

In the study, through circRNAs-sequencing identification and qRT-PCR verification, we demonstrated that circFGGY was downregulated in HCC and associated with the poor prognosis of HCC. The expression and clinical significance of circFGGY was reported in gastric cancer with no mechanistic insight. Lu et al. found that circFGGY was expressed at low level in gastric cancer and associated with the poor prognosis of patients (Lu et al., 2017). However, the role of circFGGY in HCC or other cancers remains largely unknown. In this study, we demonstrated that circFGGY had the basic characteristics of circRNA: high stability and circular structure. CircFGGY was derived from the exon 3, 4, and 5 of FGGY. As the host gene of circFGGY, it was recently reported that FGGY was a novel tumor suppressor gene and disruption of FGGY gene could promote the development and progression of lung squamous cell carcinoma (Zhang et al., 2019). However, the expression of FGGY did not change significantly upon overexpression or silencing of circFGGY. We believed that the function of circFGGY was not conducted by regulating FGGY.

We confirmed that circFGGY can inhibit the proliferation, invasion and EMT process in HCC by a series of functional experiments. CircFGGY inhibited HCC cell growth and invasion both *in vitro* and *in vivo*, suggesting that circFGGY acted as a tumor suppressor in HCC.

TGF-β signaling pathway has a role in the tumorigenesis of HCC. It regulated EMT, tumor invasion and metastasis (Sun et al., 2017). We found that the downstream molecule of TGFβ signaling pathway Smad7 was regulated by circFGGY at both the mRNA and protein level. Smad7, as a vital inhibitor of the TGFβ signaling pathway, can suppress the progression of HCC, and its deficiency accelerates tumorigenesis of HCC (Wang et al., 2013).

Previous studies suggested that circRNAs primarily function as miRNA sponges to regulate gene expression (Huang et al., 2020; Lin et al., 2021; Ren et al., 2021). By bioinformatics prediction, we identified miR-545-3p as the common target of circFGGY and Smad7. miR-545-3p is a tumor promoter which promotes cell proliferation, invasion and migration in HCC(Changjun et al., 2018). The *in vivo* and *in vitro* rescue assays in this study showed that miR-545-3p alleviated the inhibition in proliferation and metastasis of HCC cells caused by circFGGY overexpression. Moreover, circFGGY can promote the mRNA and protein expression of Smad7 and reduce the level of EMT biomarkers and these processes were rescued by miR-545-3p.

In summary, our data indicate that circFGGY is downregulated in HCC. Upregulation of circFGGY contributes to the suppression of proliferation, invasion and EMT of HCC *via* acting as a miRNAs sponge to target miR-545-3p and Smad7.

## Data Availability

The original contributions presented in the study are included in the article/Supplementary Material, further inquiries can be directed to the corresponding author

## Supplementary Material

Supplementary Figure S1 The transfection efficiency of circFGGY plasmids and ASO. **(A)** MHCC97H cells transfected with the circFGGY overexpression vector or circFGGY NC-vector and HepG2 cells transfected with ASO-NC were observed by fluorescence microscope. Scale bar, 50 µm. **(B)** qRT-PCR validated the upregulation of circFGGY in MHCC97H cells transfected with circFGGY overexpression vector and downregulation in HepG2 cells transfected with ASO-circFGGY (*n* = 3). **(C)** Statistical diagram of Figure 3E (*n* = 3). **(D)** Statistical diagram of Figure 3J (*n* = 5). Data were shown as mean ± SD. *: *p* < 0.05, **: *p* < 0.01 in independent Student’s t test.

Supplementary Figure S2 The RNA expression of putative targets after regulating circFGGY. **(A)** qRT-PCR detected the expression of smad2, smad3, smad4, smad6 and FGGY in MHCC97H cells transfected with circFGGY overexpression vector (*n* = 4). **(B)** Statistical diagram of Figure 4B. **(C)** qRT-PCR detected the expression of miR-545-3p in MHCC97H cells transfected with circFGGY overexpression vector and in HepG2 cells transfected with ASO-circFGGY (*n* = 3). **(D)** qRT-PCR detected the expression of circFGGY in HepG2 cells transfected with miR-545-3p mimics or inhibitors (*n* = 3). **(E)** Statistical diagram of Figure 7E (*n* = 5). Data are presented as mean ± SD. P values were calculated by unpaired independent Student’s t test **(A–D)** and One-Way ANOVA **(E)**, *: *p* < 0.05; **: *p* < 0.01; ***: *p* < 0.001.

## References

[B1] Benson A. B. D'Angelica M. I. Abbott D. E. Anaya D. A. Anders R. Are C. (2021). Hepatobiliary Cancers, Version 2.2021, NCCN Clinical Practice Guidelines in Oncology. J. Natl. Compr. Canc Netw. 19 (5), 541–565. 10.6004/jnccn.2021.0022 34030131

[B2] Changjun L. Feizhou H. Dezhen P. Zhao L. Xianhai M. (2018). MiR-545-3p/MT1M axis Regulates Cell Proliferation, Invasion and Migration in Hepatocellular Carcinoma. Biomed. Pharmacother. 108, 347–354. 10.1016/j.biopha.2018.09.009 30227328

[B3] Chen J. Yang X. Liu R. Wen C. Wang H. Huang L. (2020). Circular RNA GLIS2 Promotes Colorectal Cancer Cell Motility via Activation of the NF-Κb Pathway. Cell Death Dis. 11 (9), 788. 10.1038/s41419-020-02989-7 32968054 PMC7511409

[B4] Chen R.-X. Chen X. Xia L.-P. Zhang J.-X. Pan Z.-Z. Ma X.-D. (2019). N(6)-methyladenosine Modification of circNSUN2 Facilitates Cytoplasmic Export and Stabilizes HMGA2 to Promote Colorectal Liver Metastasis. Nat. Commun. 10 (1), 4695. 10.1038/s41467-019-12651-2 31619685 PMC6795808

[B5] Du B. Shim J. S. (2016). Targeting Epithelial-Mesenchymal Transition (EMT) to Overcome Drug Resistance in Cancer. Molecules 21, 965. 10.3390/molecules21070965 PMC6273543 27455225

[B6] Du W. W. Yang W. Li X. Awan F. M. Yang Z. Fang L. (2018). A Circular RNA Circ-DNMT1 Enhances Breast Cancer Progression by Activating Autophagy. Oncogene 37 (44), 5829–5842. 10.1038/s41388-018-0369-y 29973691

[B7] Farinati F. Marino D. De Giorgio M. Baldan A. Cantarini M. Cursaro C. (2006). Diagnostic and Prognostic Role of Alpha-Fetoprotein in Hepatocellular Carcinoma: Both or Neither? Am. J. Gastroenterol. 101 (3), 524–532. 10.1111/j.1572-0241.2006.00443.x 16542289

[B8] Glažar P. Papavasileiou P. Rajewsky N. (2014). CircBase: A Database for Circular RNAs. RNA 20 (11), 1666–1670. 10.1261/rna.043687.113 25234927 PMC4201819

[B9] Gonzalez D. M. Medici D. (2014). Signaling Mechanisms of the Epithelial-Mesenchymal Transition. Sci. Signal. 7 (344), e8. 10.1126/scisignal.2005189 PMC4372086 25249658

[B10] Han K. Wang F.-W. Cao C.-H. Ling H. Chen J.-W. Chen R.-X. (2020). CircLONP2 Enhances Colorectal Carcinoma Invasion and Metastasis through Modulating the Maturation and Exosomal Dissemination of microRNA-17. Mol. Cancer. 19 (1), 60. 10.1186/s12943-020-01184-8 32188489 PMC7079398

[B11] Hu Z. Q. Zhou S. L. Li J. Zhou Z. J. Wang P. C. Xin H. Y. (2020). Circular RNA Sequencing Identifies CircASAP1 as a Key Regulator in Hepatocellular Carcinoma Metastasis. Hepatology 72 (3), 906–922. 10.1002/hep.31068 31838741

[B12] Huang G. Liang M. Liu H. Huang J. Li P. Wang C. (2020). CircRNA hsa_circRNA_104348 Promotes Hepatocellular Carcinoma Progression through Modulating miR-187-3p/RTKN2 axis and Activating Wnt/β-Catenin Pathway. Cell Death Dis. 11 (12), 1065. 10.1038/s41419-020-03276-1 33311442 PMC7734058

[B13] Jiang Y. Chen X. Zhang W. (2021). Overexpression-based Detection of Translatable Circular RNAs Is Vulnerable to Coexistent Linear RNA Byproducts. Biochem. Biophysical Res. Commun. 558, 189–195. 10.1016/j.bbrc.2021.04.044 33940551

[B14] Kristensen L. S. Hansen T. B. Venø M. T. Kjems J. (2018). Circular RNAs in Cancer: Opportunities and Challenges in the Field. Oncogene 37 (5), 555–565. 10.1038/onc.2017.361 28991235 PMC5799710

[B15] Lamouille S. Xu J. Derynck R. (2014). Molecular Mechanisms of Epithelial-Mesenchymal Transition. Nat. Rev. Mol. Cell Biol. 15 (3), 178–196. 10.1038/nrm3758 24556840 PMC4240281

[B16] Li B. Zhu L. Lu C. Wang C. Wang H. Jin H. (2021). CircNDUFB2 Inhibits Non-small Cell Lung Cancer Progression via Destabilizing IGF2BPs and Activating Anti-tumor Immunity. Nat. Commun. 12 (1), 295. 10.1038/s41467-020-20527-z 33436560 PMC7804955

[B17] Li L. Chen J. Chen X. Tang J. Guo H. Wang X. (2016). Serum miRNAs as Predictive and Preventive Biomarker for Pre-clinical Hepatocellular Carcinoma. Cancer Lett. 373 (2), 234–240. 10.1016/j.canlet.2016.01.028 26850373 PMC6594104

[B18] Li W. Yang F.-Q. Sun C.-M. Huang J.-H. Zhang H.-M. Li X. (2020). CircPRRC2A Promotes Angiogenesis and Metastasis through Epithelial-Mesenchymal Transition and Upregulates TRPM3 in Renal Cell Carcinoma. Theranostics 10 (10), 4395–4409. 10.7150/thno.43239 32292503 PMC7150475

[B19] Li X. Yang L. Chen L.-L. (2018). The Biogenesis, Functions, and Challenges of Circular RNAs. Mol. Cell 71 (3), 428–442. 10.1016/j.molcel.2018.06.034 30057200

[B20] Liang W.-C. Wong C.-W. Liang P.-P. Shi M. Cao Y. Rao S.-T. (2019). Translation of the Circular RNA Circβ-Catenin Promotes Liver Cancer Cell Growth through Activation of the Wnt Pathway. Genome Biol. 20 (1), 84. 10.1186/s13059-019-1685-4 31027518 PMC6486691

[B21] Lin Y. Zheng Z.-H. Wang J.-X. Zhao Z. Peng T.-Y. (2021). Tumor Cell-Derived Exosomal Circ-0072088 Suppresses Migration and Invasion of Hepatic Carcinoma Cells through Regulating MMP-16. Front. Cell Dev. Biol. 9, 726323. 10.3389/fcell.2021.726323 34568335 PMC8458752

[B22] Lu R. Shao Y. Ye G. Xiao B. Guo J. (2017). Low Expression of Hsa_circ_0006633 in Human Gastric Cancer and its Clinical Significances. Tumour Biol. 39 (6), 101042831770417. 10.1177/1010428317704175 28656881

[B23] Ren Z. Yang Q. Guo J. Huang H. Li B. Yang Z. (2021). Circular RNA Hsa_circ_0000073 Enhances Osteosarcoma Cells Malignant Behavior by Sponging miR-1252-5p and Modulating CCNE2 and MDM2. Front. Cell Dev. Biol. 9, 714601. 10.3389/fcell.2021.714601 34568326 PMC8459753

[B24] Smith A. L. Gjoka E. Izhar M. Novo K. J. Mason B. C. De Las Casas A. (2021). FGGY Carbohydrate Kinase Domain Containing Is Expressed and Alternatively Spliced in Skeletal Muscle and Attenuates MAP Kinase and Akt Signaling. Gene 800, 145836. 10.1016/j.gene.2021.145836 34280510

[B25] Sun H. Peng Z. Tang H. Xie D. Jia Z. Zhong L. (2017). Loss of KLF4 and Consequential Downregulation of Smad7 Exacerbate Oncogenic TGF-β Signaling in and Promote Progression of Hepatocellular Carcinoma. Oncogene 36 (21), 2957–2968. 10.1038/onc.2016.447 28192402 PMC5444978

[B26] Sung H. Ferlay J. Siegel R. L. Laversanne M. Soerjomataram I. Jemal A. (2021). Global Cancer Statistics 2020: GLOBOCAN Estimates of Incidence and Mortality Worldwide for 36 Cancers in 185 Countries. CA A Cancer J. Clin. 71 (3), 209–249. 10.3322/caac.21660 33538338

[B27] Taylor J. A. Shioda K. Mitsunaga S. Yawata S. Angle B. M. Nagel S. C. (2018). Prenatal Exposure to Bisphenol a Disrupts Naturally Occurring Bimodal DNA Methylation at Proximal Promoter of Fggy, an Obesity-Relevant Gene Encoding a Carbohydrate Kinase, in Gonadal White Adipose Tissues of CD-1 Mice. Endocrinology 159 (2), 779–794. 10.1210/en.2017-00711 29220483 PMC5774244

[B28] Wang J. Zhao J. Chu E. S. Mok M. T. Go M. Y. Man K. (2013). Inhibitory Role of Smad7 in Hepatocarcinogenesis in Mice and *In Vitro* . J. Pathol. 230 (4), 441–452. 10.1002/path.4206 23625826

[B29] Wang J. Zhou F. Li Y. Li Q. Wu Z. Yu L. (2017). Cdc20 Overexpression Is Involved in Temozolomide-Resistant Glioma Cells with Epithelial-Mesenchymal Transition. Cell Cycle 16 (24), 2355–2365. 10.1080/15384101.2017.1388972 29108461 PMC5788407

[B30] Wang L. Long H. Zheng Q. Bo X. Xiao X. Li B. (2019). Circular RNA circRHOT1 Promotes Hepatocellular Carcinoma Progression by Initiation of NR2F6 Expression. Mol. Cancer. 18 (1), 119. 10.1186/s12943-019-1046-7 31324186 PMC6639939

[B31] Wang S. Zhang K. Tan S. Xin J. Yuan Q. Xu H. (2021). Circular RNAs in Body Fluids as Cancer Biomarkers: The New Frontier of Liquid Biopsies. Mol. Cancer 20 (1), 13. 10.1186/s12943-020-01298-z 33430880 PMC7798340

[B32] Wei Y. Chen X. Liang C. Ling Y. Yang X. Ye X. (2020). A Noncoding Regulatory RNAs Network Driven by Circ‐CDYL Acts Specifically in the Early Stages Hepatocellular Carcinoma. Hepatology 71 (1), 130–147. 10.1002/hep.30795 31148183

[B33] Wu A. Hu Y. Xu Y. Xu J. Wang X. Cai A. (2021). Methyltransferase-Like 3-Mediated m6A Methylation of Hsa_circ_0058493 Accelerates Hepatocellular Carcinoma Progression by Binding to YTH Domain-Containing Protein 1. Front. Cell Dev. Biol. 9, 762588. 10.3389/fcell.2021.762588 34888309 PMC8650312

[B34] Wu N. Xu J. Du W. W. Li X. Awan F. M. Li F. (2021). YAP Circular RNA, circYap, Attenuates Cardiac Fibrosis via Binding with Tropomyosin-4 and Gamma-Actin Decreasing Actin Polymerization. Mol. Ther. 29 (3), 1138–1150. 10.1016/j.ymthe.2020.12.004 33279723 PMC7934790

[B35] Yang J. Weinberg R. A. (2008). Epithelial-mesenchymal Transition: At the Crossroads of Development and Tumor Metastasis. Dev. Cell 14 (6), 818–829. 10.1016/j.devcel.2008.05.009 18539112

[B36] Yu J. Xu Q.-g. Wang Z.-g. Yang Y. Zhang L. Ma J.-z. (2018). Circular RNA cSMARCA5 Inhibits Growth and Metastasis in Hepatocellular Carcinoma. J. Hepatology 68 (6), 1214–1227. 10.1016/j.jhep.2018.01.012 29378234

[B37] Zhang R. Zhang F. Sun Z. Liu P. Zhang X. Ye Y. (2019). LINE-1 Retrotransposition Promotes the Development and Progression of Lung Squamous Cell Carcinoma by Disrupting the Tumor-Suppressor Gene FGGY. Cancer Res. 79 (17), 4453–4465. 10.1158/0008-5472.CAN-19-0076 31289132

[B38] Zhou W. Gong L. Wu Q. Xing C. Wei B. Chen T. (2018). PHF8 Upregulation Contributes to Autophagic Degradation of E-Cadherin, Epithelial-Mesenchymal Transition and Metastasis in Hepatocellular Carcinoma. J. Exp. Clin. Cancer Res. 37 (1), 215. 10.1186/s13046-018-0890-4 30180906 PMC6122561

[B39] Zhu Y.-J. Zheng B. Luo G.-J. Ma X.-K. Lu X.-Y. Lin X.-M. (2019). Circular RNAs Negatively Regulate Cancer Stem Cells by Physically Binding FMRP against CCAR1 Complex in Hepatocellular Carcinoma. Theranostics 9 (12), 3526–3540. 10.7150/thno.32796 31281495 PMC6587157

