# Supplementary figures and images for: CircFGGY Inhibits Cell Growth, Invasion and Epithelial-Mesenchymal Transition of Hepatocellular Carcinoma via Regulating the miR-545-3p/Smad7 Axis
Source: Front Cell Dev Biol. 2022 May 3;10:850708. doi: 10.3389/fcell.2022.850708 (PMC9110866; doi:10.3389/fcell.2022.850708)

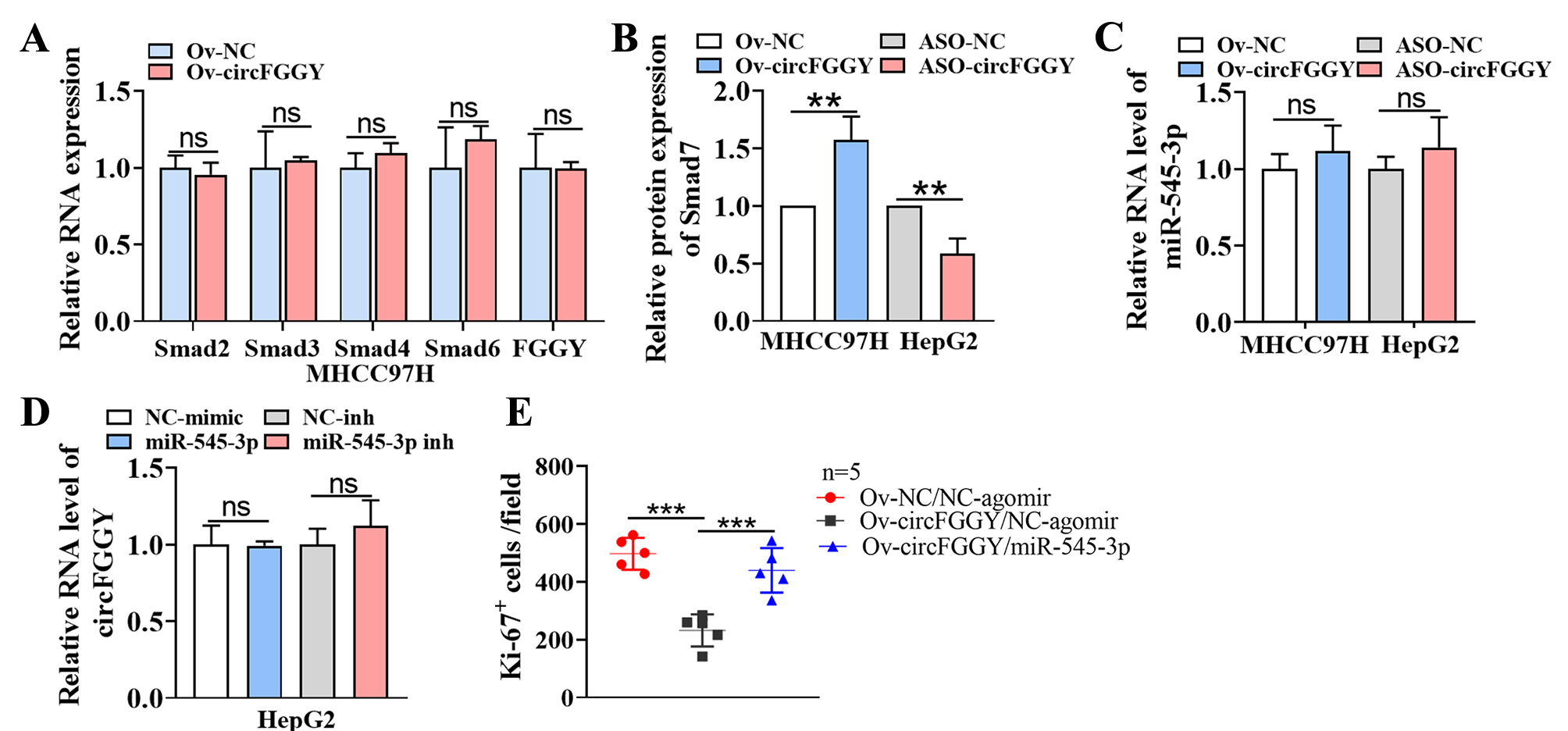

Supplement: Supplementary file 3 [file Image2.TIF]

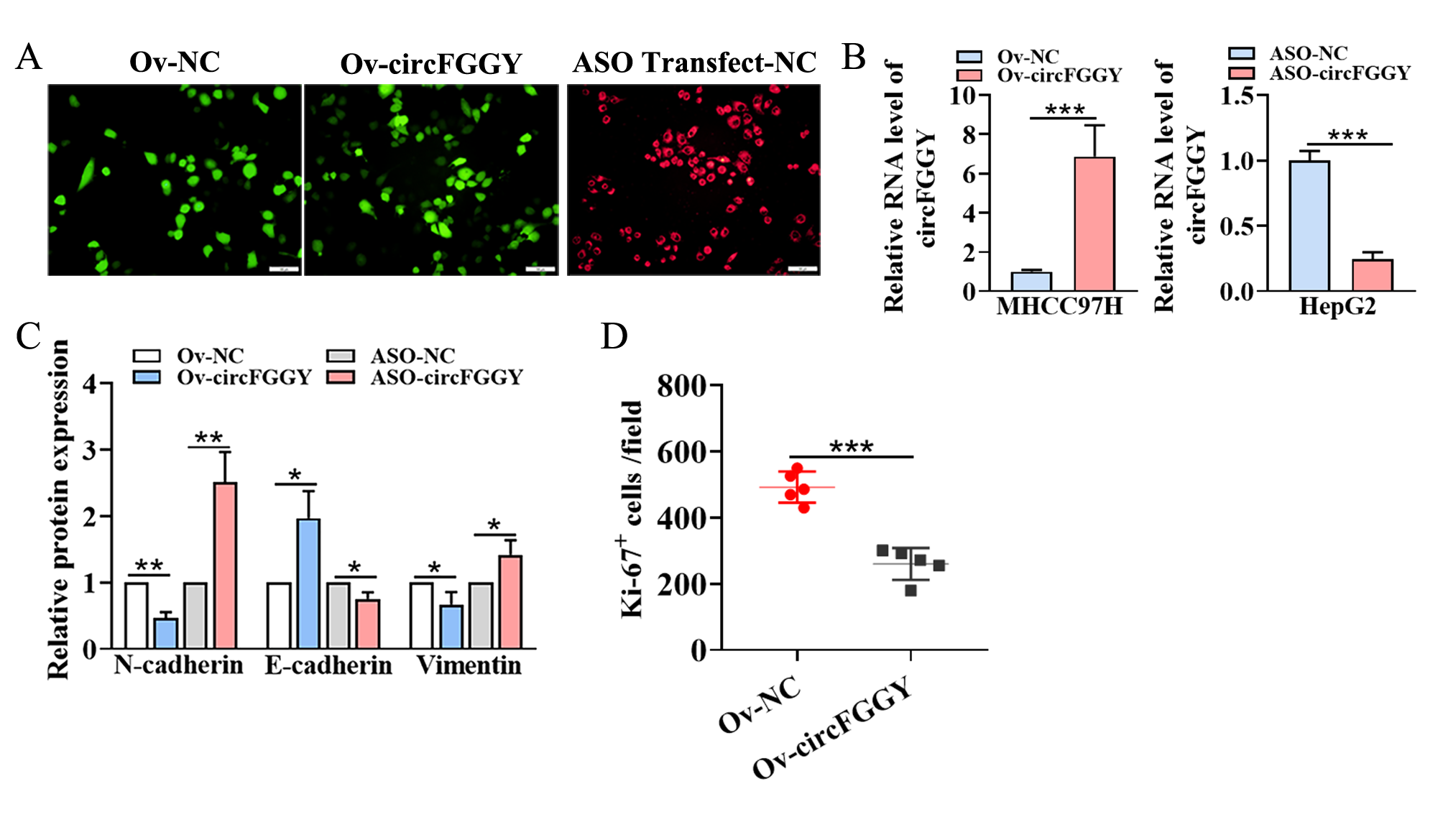

Supplement: Supplementary file 4 [file Image1.TIF]
